# Effects of Circadian Phase and Exercise Training on Systemic and Tissue-Specific Redox Responses in Mice Fed a High-Fat Diet

**DOI:** 10.3390/nu18203384

**Published:** 2026-10-15

**Authors:** Shuo Wang, Ziwei Zhang, Jiapeng Huang, Yishan Tong, Cong Wu, Haruki Kobori, Yasuhiro Seki, Sihui Ma, Katsuhiko Suzuki

**Affiliations:** 1 Graduate School of Sport Sciences, Waseda University, Tokorozawa 359-1192, Japan; 2 Faculty of Sport Sciences, Waseda University, Tokorozawa 359-1192, Japan

**Keywords:** circadian rhythm, exercise timing, chrono-exercise, oxidative stress, antioxidant capacity, skeletal muscle, high-fat diet

## Abstract

Background: The effects of exercise timing on redox adaptation under high-fat diet (HFD)-induced metabolic stress remain unclear. We investigated whether the circadian phase of training alters systemic and tissue-specific redox responses in HFD-fed mice. Methods: Male C57BL/6J mice fed an HFD remained sedentary or underwent 8 weeks of treadmill training at Zeitgeber time 3 (ZT3) or Zeitgeber time 15 (ZT15). Samples were collected 48 h after the final session. Plasma redox status was assessed using the OXY-adsorbent test (OXY), the derivatives of reactive oxygen metabolites test (d-ROMs; mainly reflecting circulating hydroperoxides), and the biological antioxidant potential test (BAP), and redox and inflammatory gene expression levels were quantified in three skeletal muscles and the liver. Results: Exercise increased BAP (*p* = 0.0442), whereas OXY was unchanged. The phase × exercise interaction for d-ROMs showed a nonsignificant trend (*p* = 0.0560); exploratory comparisons showed higher d-ROMs in ZT15-sedentary (sed) than in ZT3-sed and ZT15-exercise (exe) mice. Gastrocnemius (GAS) *Nfe2l2* showed a nominal phase × exercise interaction. Soleus (SOL) *Hmox1* was higher at ZT15 and with exercise. Quadriceps (QUA) *Nfe2l2* was higher at ZT3 and *Cat* at ZT15. Hepatic *Keap1* was higher at ZT15, particularly in sedentary mice, while inflammatory transcripts remained largely unchanged. Only the phase effects on SOL *Hmox1* and hepatic *Keap1* remained significant after false discovery rate (FDR) correction. Conclusions: Long-term exercise was associated with higher plasma ferric-reducing capacity irrespective of circadian phase, whereas tissue redox-related transcriptional responses were mainly associated with circadian phase and differed among tissues. Whether active-phase exercise provides a redox advantage over rest-phase exercise requires further investigation.

## 1. Introduction

Regular physical exercise plays a pivotal role in maintaining metabolic homeostasis and redox balance. A substantial body of evidence demonstrates that long-term exercise training enhances antioxidant capacity [1], mitigates inflammatory responses, and improves overall metabolic health. However, the adaptive effects of exercise are not constant but are modulated by multiple factors, including exercise intensity, duration, and circadian phase. Among these, exercise timing has recently been recognized as a critical chronobiological determinant of metabolic responses and training efficiency [2,3]. These observations raise the possibility that the redox adaptations induced by long-term exercise may also depend on the circadian phase at which exercise is performed.

Circadian regulation provides a biological basis for time-dependent differences in exercise-induced redox responses. In mammals, central and peripheral circadian oscillators exhibit coordinated but tissue-dependent rhythms that contribute to the temporal organization of physiological and metabolic processes [4,5]. At the molecular level, these rhythms are generated by interlocked transcription–translation feedback loops [6,7]. In the core loop, the transcription factors circadian locomotor output cycles kaput (CLOCK) and brain and muscle ARNT-like 1 (BMAL1) form a heterodimer that binds E-box elements and activates the transcription of the *Period* (*Per1/2*) and *Cryptochrome* (*Cry1/2*) genes [7,8]. PER and CRY proteins then accumulate, enter the nucleus, and repress CLOCK–BMAL1 activity, thereby completing a cycle of approximately 24 h [7]. In an auxiliary loop, CLOCK–BMAL1 also induces *Nr1d1*, which encodes the nuclear receptor REV-ERBα, and REV-ERBα in turn represses *Bmal1* transcription [9]. Beyond these core components, CLOCK–BMAL1 drives the rhythmic expression of numerous clock-controlled genes that regulate energy metabolism, redox balance, and hormonal secretion [6]; accordingly, mice carrying a *Clock* mutation develop obesity and features of the metabolic syndrome [10]. Metabolically active organs, including skeletal muscle, liver, and adipose tissue, all express functional circadian clocks. In skeletal muscle, the integrity of the internal clock contributes to the regulation of glucose metabolism, muscle function, and systemic inflammation [11], and mice lacking functional CLOCK or BMAL1 show reduced muscle force and reduced mitochondrial volume [12]. Disruption of circadian rhythms or sleep has been shown to elevate oxidative stress and impair muscle function, whereas regular physical activity might alter the temporal pattern of clock gene expression [13] and enhance antioxidant defense [1]. Moreover, antioxidant-related genes such as *Nfe2l2* and *Hmox1* exhibit pronounced circadian oscillations across tissues [14,15], and *Nfe2l2* is directly regulated by CLOCK–BMAL1, linking the core clock to antioxidant defense [14]. Consequently, identical exercise stimuli administered at different times of the day may elicit distinct redox and antioxidant responses.

Exercise-induced reactive oxygen species (ROS) signaling represents a direct link between physical activity and redox adaptation. Rather than functioning solely as metabolic by-products, ROS generated during exercise can participate in redox-sensitive signaling that mediates exercise adaptation [16,17,18]. When maintained within a physiological range—sometimes described as a “Goldilocks Zone”—ROS can promote oxidative eustress and contribute to adaptive processes such as mitochondrial biogenesis [16,17]. This range is not defined by a fixed concentration; it depends on the type, source, subcellular location, magnitude, and duration of ROS production and is generally inferred from whether adaptive signaling or oxidative damage predominates [16,17]. Moderate ROS production can initiate adaptive redox responses involving nuclear factor erythroid 2-related factor 2 (NRF2)-mediated signaling, thereby promoting the expression of antioxidant and cytoprotective enzymes including heme oxygenase-1 (HO-1), NAD(P)H:quinone oxidoreductase 1 (NQO1), superoxide dismutase 2 (SOD2), catalase (CAT), and glutathione peroxidase 1 (GPX1) [1,19]. This redox-sensitive signaling is highly compartmentalized, requiring precise spatiotemporal coordination to prevent the transition from beneficial adaptation to detrimental oxidative or reductive stress [20,21]. In contrast, excessive ROS accumulation can damage cellular structures and impair metabolic and contractile functions under conditions of insufficient antioxidant protection. Antioxidant enzyme activities and their responses to exercise differ among skeletal muscles with different fiber-type compositions, suggesting that exercise-induced redox responses may be muscle-specific [22]. Such heterogeneity may contribute to a muscle-specific “redox landscape” that influences skeletal muscle function and systemic resilience [23]. Therefore, the efficiency of exercise-induced adaptation and recovery depends on the coordinated regulation of ROS generation and antioxidant defense.

The circadian clock and nutrient metabolism are closely interconnected. BMAL1 promotes adipogenesis and lipid storage [24], REV-ERBα/β coordinate hepatic lipid metabolism [25], and the intrinsic skeletal muscle clock regulates muscle insulin sensitivity and glucose metabolism [26]. Conversely, high-fat diet (HFD) feeding dampens behavioral and molecular circadian rhythms [27]. In our previous study of the same cohort, hepatic triglyceride content and *Cpt1a* expression differed between Zeitgeber time 3 (ZT3) and Zeitgeber time 15 (ZT15) [28]. Because fatty acid oxidation is an important source of mitochondrial ROS, such phase-dependent metabolic states may also influence redox responses to exercise. Metabolic stress may further modify the relationship between exercise timing and redox regulation. The interaction between exercise timing and oxidative stress responses remains incompletely understood, particularly under the metabolic stress imposed by an HFD. Nutritional stressors such as an HFD can induce extensive transcriptomic reprogramming and disrupt the synergy between core clock oscillations and redox signaling [29,30]. Such disruption may alter both systemic oxidative stress and tissue-specific antioxidant responses to exercise. Most existing studies have focused on acute exercise-induced oxidative stress, whereas the effects of long-term, time-specific exercise training on systemic redox status and antioxidant and inflammatory gene expression across different skeletal muscles and the liver remain largely unexplored. Clarifying these responses may contribute to the optimization of exercise timing for populations with obesity and metabolic dysfunction.

In nocturnal mice, ZT3 corresponds to the early rest (light) phase and ZT15 to the early active (dark) phase, and these time points have been widely used to compare rest-phase and active-phase exercise [2,31]. In conventional rodent exercise studies, training is frequently performed during the light phase, which coincides with the rest phase of nocturnal animals [32]; however, long-term training performed during the rest phase has rarely been compared directly with active-phase training [33,34,35]. Relative to the environmental light–dark cycle, ZT3 and ZT15 correspond to approximately 3 h after dawn and 3 h after dusk, respectively, time windows commonly used for human exercise in daily life, and human studies have reported that morning and evening exercise elicit different autonomic and metabolic responses [36,37]. Because mice are nocturnal, the same environmental time corresponds to opposite internal circadian phases in mice and humans; the ZT3–ZT15 contrast therefore addresses whether the internal circadian phase at which training occurs modifies exercise-induced adaptations.

Accordingly, the present study investigated whether long-term exercise training performed at ZT3 or ZT15 differentially affects systemic and tissue-specific redox responses in HFD-fed mice. Using an established HFD exercise-timing model [28], we assessed plasma oxidant load and antioxidant capacity, and redox and inflammatory gene expression in three skeletal muscles with different fiber-type compositions and the liver. Given the limited evidence in this area, the present study was designed as an exploratory, hypothesis-generating investigation. We hypothesized that exercise-induced redox responses would vary according to circadian phase and tissue type.

## 2. Materials and Methods

### 2.1. Experimental Protocol

The analyses used a previously described cohort of 32 seven-week-old male C57BL/6J mice obtained from Takasugi Experimental Animal Supply Co., Ltd. (Kasukabe, Japan) and acclimated for 2 weeks [28]. The previous publication reported lipid metabolic outcomes, whereas the plasma redox markers and tissue gene expression outcomes examined in the present study have not been reported previously. No single primary outcome was prespecified for the present redox-focused analyses. The study used a 2 × 2 factorial design with two factors: circadian phase (ZT3 or ZT15) and exercise condition (sedentary or exercise). This animal experiment was reviewed and approved by the Institutional Animal Care and Use Committee of Waseda University (approval number A23-126; approved 11 April 2023), and the investigation was conducted and described following the Animal Research: Reporting of In Vivo Experiments (ARRIVE) 2.0 guidelines.

### 2.2. Animals and Circadian Phase Manipulation (Figure 1)

The circadian phase manipulation protocol was performed as described in our previous study [28]. Briefly, mice were housed under a 12-h light/dark cycle (lights on at 08:00 and off at 20:00; ZT0 = 08:00) and fed an HFD (D12492; Research Diets, Inc., New Brunswick, NJ, USA; 60 kcal% fat) *ad libitum*. Two circadian phase groups were established: ZT3 (*n* = 16), maintained on the standard light–dark cycle; and ZT15 (*n* = 16), which underwent 24 h of constant light on the third day of HFD feeding (Day 1) followed by a 12-h light–dark reversal (lights on at 20:00 and off at 08:00; new ZT0 = 20:00), leading to a sustained 12-h delay in circadian phase without disrupting the 24-h cycle [38,39]. Accordingly, 11:00 local time corresponded to ZT3 in the ZT3 group and to ZT15 in the ZT15 group, allowing both groups to be trained at the same clock time. Re-entrainment of the ZT15 group was confirmed by the active/rest feeding ratio, which stabilized approximately 12 days after the reversal [28].

**Figure 1:**
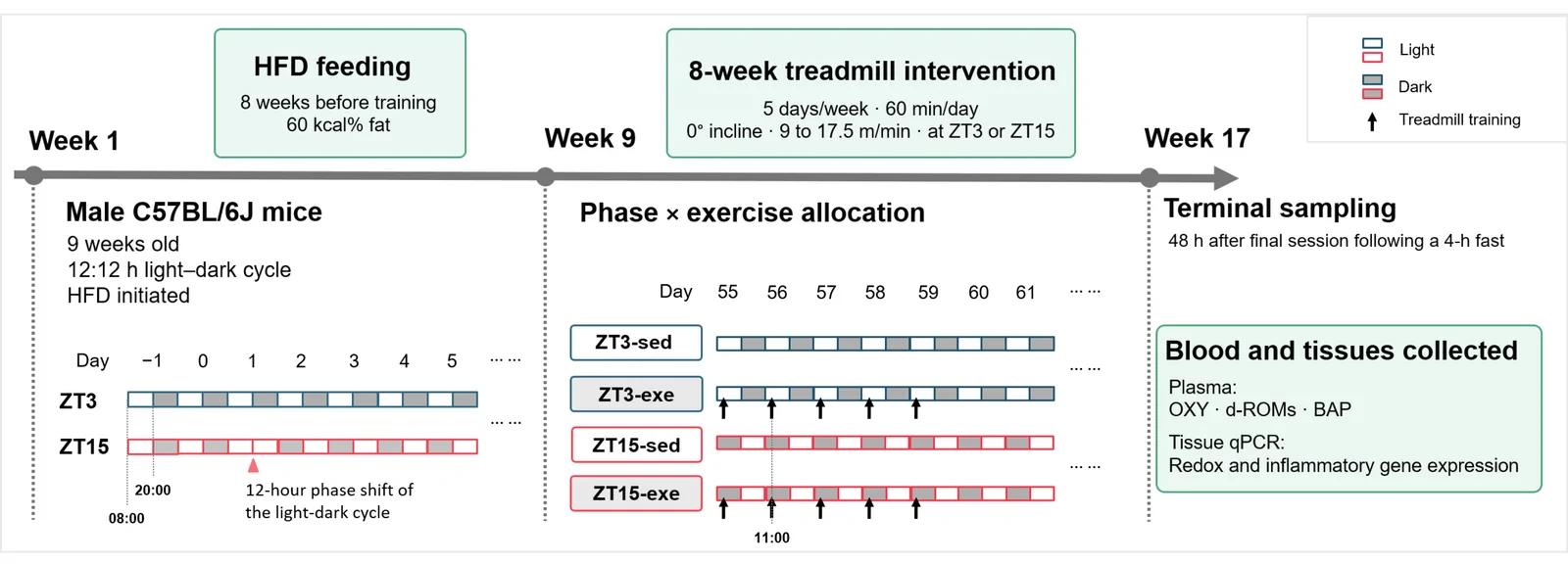
Experimental design and outcome measurements. Male C57BL/6J mice received a high-fat diet (HFD; 60 kcal% fat) for an initial 8-week period and were subsequently allocated to either sedentary or treadmill exercise groups at Zeitgeber time 3 (ZT3) or 15 (ZT15). On the third day of HFD feeding (Day 1 in the figure), the ZT15 group was exposed to 24 h of constant light followed by a reversed light–dark cycle. The HFD was continued throughout the 8-week exercise intervention (Weeks 9–16). Blood and tissue samples were collected after a 4-h fast, 48 h after the last exercise bout. Training was performed at 11:00 local time in both groups, corresponding to ZT3 in the ZT3 group and ZT15 in the ZT15 group. Plasma redox markers and redox and inflammatory gene expression in skeletal muscle and liver were analyzed. Final group sizes were ZT3-sed, *n* = 5; ZT3-exe, *n* = 8; ZT15-sed, *n* = 8; and ZT15-exe, *n* = 8 (sed, sedentary; exe, exercise). The same experimental cohort was used in our previous study [28].

After the initial 8-week HFD period, mice within each phase condition were allocated to sedentary or exercise subgroups (ZT3-sed, ZT3-exe, ZT15-sed, and ZT15-exe; sed, sedentary; exe, exercise; *n* = 8 per group before exclusions) by pairwise randomization according to body weight. Animals were excluded according to institutional animal welfare criteria when body weight loss exceeded 15% or visible injury was observed. In Week 10 (the second week of the intervention), three mice housed in the same ZT3-sed cage sustained severe fighting-related injuries. Although they were separated immediately, all three were excluded from the final dataset because wound-related inflammation could affect inflammatory and redox-related gene expression. After exclusion, the final sample sizes were ZT3-sed, *n* = 5; ZT3-exe, *n* = 8; ZT15-sed, *n* = 8; and ZT15-exe, *n* = 8, as reported previously [28]. Investigators who conducted the biochemical and molecular analyses were blinded to the group allocations.

### 2.3. Exercise Intervention and Sample Collection

Before the intervention, mice underwent a two-day treadmill familiarization period. The subsequent treadmill training protocol was performed as described previously [28]. Briefly, exercise groups (ZT3-exe, ZT15-exe) underwent progressive moderate-to-vigorous treadmill running (KN-73, Natsume Seisakusho Co., Ltd., Tokyo, Japan; 0° incline; equivalent to approximately 60–70% of maximal oxygen uptake [VO_2_max] in obese C57BL/6J mice [40,41]) for 8 weeks (5 days/week, 60 min/session, starting in Week 9 of HFD feeding) at their assigned circadian phase (11:00 local time in both groups). Training speed progressed from 9 m/min (Week 9) to a plateau of 17.5 m/min from Week 13 onward, as detailed previously [28] (Figure 1). No electric shocks were used to motivate running; mice were gently encouraged with light tail tapping when necessary. Sedentary control mice were kept close to the treadmill at the corresponding time point but did not run.

Blood and tissue samples were collected 48 h after the final exercise session, starting at 11:00 local time (ZT3 or ZT15, in the respective group). Each mouse was fasted for 4 h before sampling, which took approximately 15 min per mouse, and the sampling order was rotated among groups [28]. Approximately 700–800 μL of blood was collected from the posterior vena cava into heparinized tubes under isoflurane inhalation anesthesia (Isoflurane RIKEN; RikenVets Pharma Co., Ltd., Saitama, Japan) and centrifuged at 3000× *g* for 10 min at 4 °C. The separated plasma (approximately 300 μL) was stored at −80 °C until analysis. Mice were then euthanized by cervical dislocation while under anesthesia, in accordance with the approved protocol, and the gastrocnemius (GAS), soleus (SOL), quadriceps (QUA), and liver were collected, washed in phosphate-buffered saline to remove attached blood, gently blotted, snap-frozen in liquid nitrogen, and stored at −80 °C until RNA extraction.

### 2.4. Assessment of Oxidative Stress and Antioxidant Capacity

Plasma oxidant and antioxidant measures were determined colorimetrically using a Free Radical Elective Evaluator (FREE; Wismerll Co., Ltd., Tokyo, Japan) with the OXY-adsorbent test (OXY), derivatives of reactive oxygen metabolites test (d-ROMs), and biological antioxidant potential test (BAP), according to the manufacturer’s protocols [42,43].

The OXY test assessed the capacity of plasma to neutralize an exogenously added hypochlorous acid (HClO) challenge. Plasma was mixed with the HClO reagent, allowing plasma constituents with antioxidant activity to neutralize HClO. After a fixed reaction period, *N,N*-diethyl-*p*-phenylenediamine (DEPPD) was added. The remaining unneutralized HClO oxidized DEPPD to a colored radical cation, which was quantified spectrophotometrically at 505 nm. Results were expressed in μmol HClO/mL. The BAP test assessed the ferric-reducing capacity of plasma. A ferric chloride reagent was mixed with a chromogenic thiocyanate derivative to form a colored Fe^3+^–chromogen complex. After plasma was added, reducing substances in the sample donated electrons and converted Fe^3+^ to Fe^2+^, resulting in decolorization of the complex. The decrease in absorbance at 505 nm was proportional to the ferric-reducing capacity of the sample [44], and results were expressed in μmol/L. The d-ROMs test assessed plasma oxidant capacity, primarily reflecting hydroperoxide-derived oxidants rather than directly identifying or quantifying individual ROS [45,46]. Plasma was mixed with an acetate buffer (pH 4.8), in which transition-metal ions released from plasma proteins catalyzed the conversion of hydroperoxides (R–OOH) into alkoxyl (R–O•) and peroxyl (R–OO•) radicals through Fenton-type reactions as follows:

R–OOH + Fe^2+^ → R–O• + Fe^3+^ + OH^−^

R–OOH + Fe^3+^ → R–OO• + Fe^2+^ + H^+^

The resulting radicals oxidized DEPPD to its colored radical cation, which was measured spectrophotometrically at 505 nm. The formation of the DEPPD radical cation has been characterized by electron spin resonance [47]. Results were expressed in Carratelli units (CARR U), with 1 CARR U corresponding to an oxidant capacity equivalent to 0.08 mg H_2_O_2_/100 mL [45,47]; this value represents an H_2_O_2_ equivalent rather than the actual H_2_O_2_ concentration [48].

### 2.5. Quantitative Real-Time Polymerase Chain Reaction (qPCR)

Total RNA was extracted using the NucleoSpin RNA Kit (Takara Bio Inc., Kusatsu, Japan) and its concentration and purity were measured using a NanoDrop spectrophotometer (NanoDrop Technologies, Wilmington, DE, USA). Complementary DNA (cDNA) was synthesized using the PrimeScript^TM^ FAST RT Reagent Kit with gDNA Eraser (Takara Bio Inc., Kusatsu, Japan). qPCR was conducted using TB Green^®^ Premix Ex Taq™ II FAST qPCR on a Thermal Cycler Dice^®^ Real Time System IV (Takara Bio Inc.) according to the manufacturer’s instructions. Amplification specificity was confirmed by melting-curve analysis, which yielded a single peak for each primer pair. Candidate reference genes were evaluated for stability across the experimental groups. Among these candidates, *Gapdh* in GAS and *Rps18* in SOL, QUA, and liver showed the lowest variability across groups (coefficient of variation of cycle threshold [Ct] < 2%) and were therefore used as controls for tissue-specific normalization. Relative messenger RNA (mRNA) expression levels were calculated using the 2^−ΔΔCt^ method and presented as fold changes relative to the ZT3-sed group. Table 1 lists the primer sequences for genes investigated in the main text, whereas those for the supplementary gene-expression analyses are provided in Table S1.

### 2.6. Statistical Analysis

Data analyses were performed using GraphPad Prism 10.4.0 (GraphPad Software, La Jolla, CA, USA). Data normality and homogeneity of variance were assessed using the Shapiro–Wilk and Brown–Forsythe tests, respectively. Four-group comparisons were performed using two-way analysis of variance (ANOVA) with circadian phase (ZT3 vs. ZT15) and exercise condition (sedentary vs. exercise) as factors. When the phase × exercise interaction was significant or showed a nonsignificant trend (0.05 ≤ *p* < 0.10), exploratory pairwise comparisons of simple effects were performed using Fisher’s least significant difference test. Partial η^2^ was reported as the effect size for ANOVA, and mean differences and 95% confidence intervals (CIs) were reported for pairwise comparisons where applicable. All tests were two-tailed, with statistical significance set at *p* < 0.05. Because multiple outcomes were analyzed without a prespecified primary endpoint, *p* values were additionally adjusted using the Benjamini–Hochberg false discovery rate (FDR) procedure separately for each ANOVA term (phase, exercise, and phase × exercise) within each outcome family (plasma redox markers, the gene panel of each tissue, and the supplementary genes of each tissue). Unadjusted *p* values are reported in the text, and all FDR-adjusted *q* values are provided in Table S2. Values are presented as means ± standard error of the mean (SEM).

## 3. Results

### 3.1. Systemic Redox Status (Figure 2)

**Figure 2:**
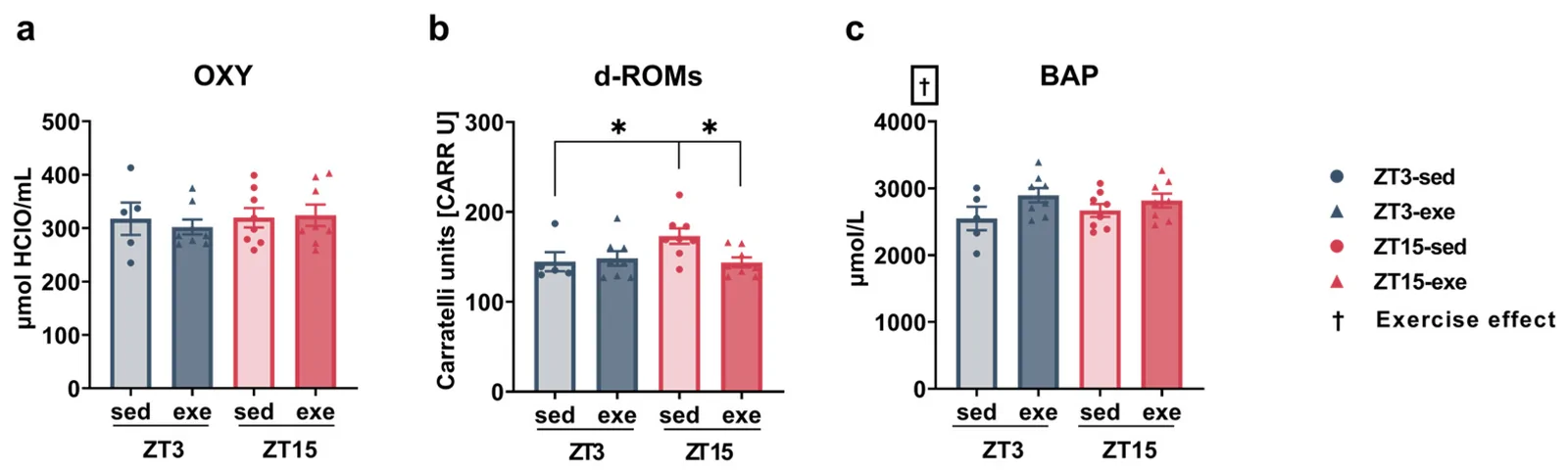
Plasma markers of oxidative stress and antioxidant capacity. (**a**) OXY-adsorbent test (OXY, μmol HClO/mL), antioxidant capacity against HClO; (**b**) derivatives of reactive oxygen metabolites test (d-ROMs, Carratelli units [CARR U]), hydroperoxide load; and (**c**) biological antioxidant potential test (BAP, μmol/L), plasma ferric-reducing capacity. Statistical analyses were performed using two-way analysis of variance (ANOVA, phase × exercise). Data are means ± standard error of the mean (SEM, ZT3-sed *n* = 5, ZT3-exe *n* = 8, ZT15-sed *n* = 8, ZT15-exe *n* = 8). † indicates an exercise main effect, and * indicates a *post hoc* comparison; †/* *p* < 0.05. Symbols indicate unadjusted *p* values; false discovery rate (FDR)-adjusted *q* values are provided in Table S2.

To determine circulating redox status in the context of exercise timing, OXY, d-ROMs, and BAP were measured (Figure 2). OXY showed no significant main effects or interactions between phase and exercise (Figure 2a). For d-ROMs, the phase × exercise interaction approached significance (*F*(1, 25) = 4.02, *p* = 0.0560, partial η^2^ = 0.14); exploratory *post hoc* comparisons indicated that d-ROMs values in the ZT15-sed group were higher than those in both the ZT3-sed (*p* = 0.0293, 95% CI [3.11, 53.94]) and ZT15-exe (*p* = 0.0122, 95% CI [6.96, 51.54]) groups (Figure 2b). BAP exhibited a significant main effect of exercise (*F*(1, 25) = 4.49, *p* = 0.0442, partial η^2^ = 0.15), indicating higher plasma reducing capacity in exercised mice (Figure 2c), although this effect did not remain significant after FDR correction (*q* = 0.13; Table S2). Overall, exercise was associated with higher BAP-assessed plasma antioxidant potential, whereas d-ROMs showed a possible phase-dependent response to exercise that should be interpreted in light of the nonsignificant interaction trend.

### 3.2. GAS Antioxidant and Inflammatory Gene Expression (Figure 3)

Among the antioxidant and redox-related genes in the GAS muscle, a significant interaction between phase and exercise was found for *Nfe2l2* (*F*(1, 25) = 4.86, *p* = 0.0370, partial η^2^ = 0.16), although this interaction did not remain significant after FDR correction (*q* = 0.30; Table S2). *Post hoc* analysis showed that *Nfe2l2* expression was higher in ZT15-sed than in ZT3-sed (*p* = 0.0478, 95% CI [0.01, 1.59]). There were no significant differences for the other antioxidant and redox-related genes (*Hmox1*, *Sod2*, *Cat*, and *Gpx1*) or for the inflammation-related genes (*Tnf*, *Il1b*, and *Il6*). Ancillary analyses of core clock gene expression are presented in Figure S1; all phase and exercise effects shown therein remained significant after FDR correction (Table S2). Overall, GAS *Nfe2l2* expression showed a nominal phase × exercise interaction, whereas no significant effects were detected for the other antioxidant, redox-related, or inflammation-related genes.

**Figure 3:**
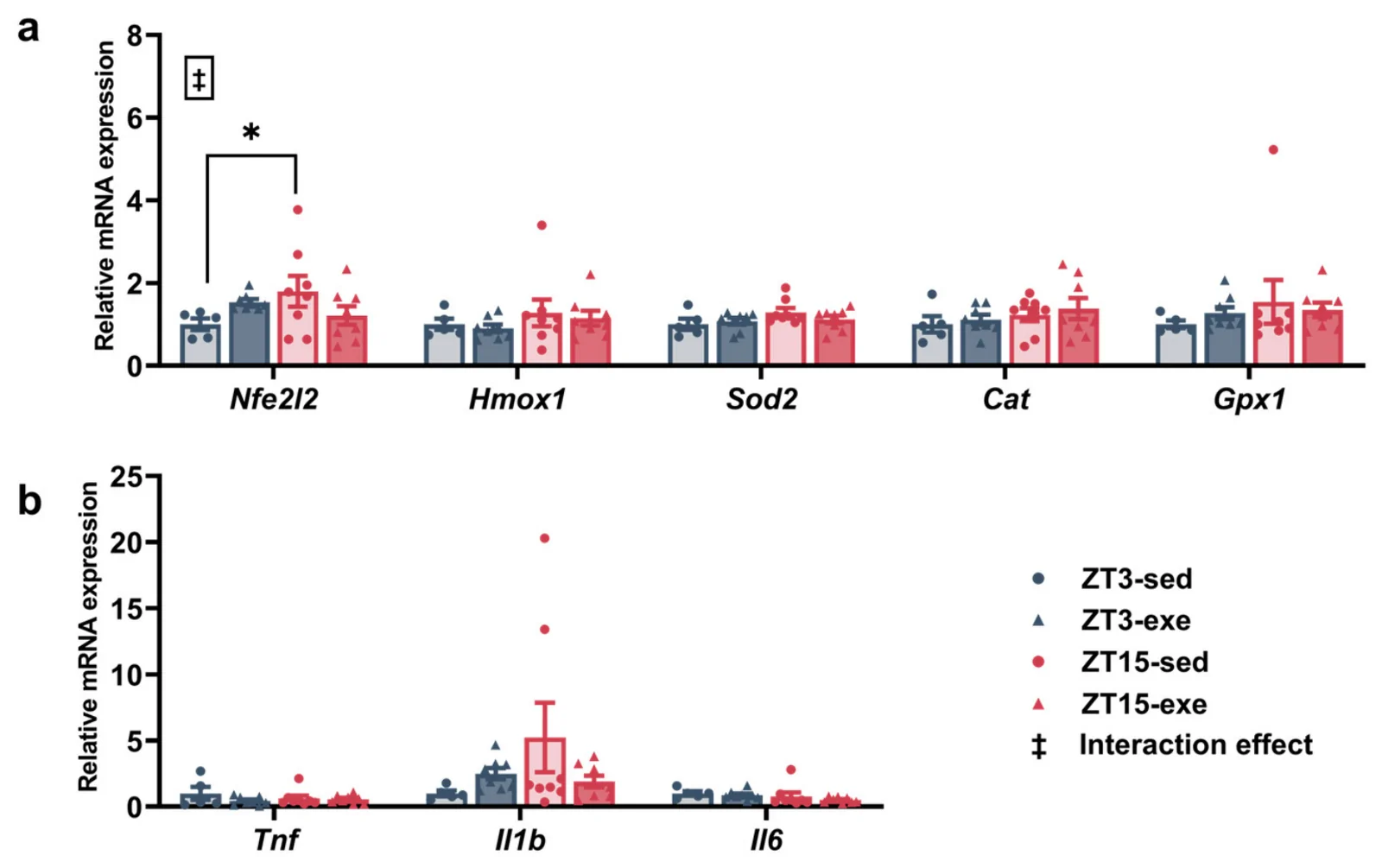
Gastrocnemius (GAS) antioxidant and inflammatory gene expression. (**a**) Antioxidant genes (*Nfe2l2*, *Hmox1*, *Sod2*, *Cat*, and *Gpx1*); and (**b**) inflammatory genes (*Tnf, Il1b*, and *Il6*). Group differences were examined using two-way ANOVA with phase and exercise as factors. Data are presented as means ± SEM (ZT3-sed *n* = 5, ZT3-exe *n* = 8, ZT15-sed *n* = 8, ZT15-exe *n* = 8). ‡ indicates a phase × exercise interaction, and * indicates a *post hoc* comparison; ‡/* *p* < 0.05. Symbols indicate unadjusted *p* values; FDR-adjusted *q* values are provided in Table S2.

### 3.3. SOL Antioxidant and Inflammatory Gene Expression (Figure 4)

In the SOL muscle, *Hmox1* was significantly modulated by both phase (*F*(1, 25) = 8.36, *p* = 0.0078, partial η^2^ = 0.25) and exercise status (*F*(1, 25) = 6.32, *p* = 0.0187, partial η^2^ = 0.20), with higher expression in ZT15 than ZT3 mice and in exercised than in sedentary mice. After FDR correction, the phase effect remained significant (*q* = 0.039), whereas the exercise effect did not (*q* = 0.094; Table S2). *Cat* showed a nonsignificant trend toward a phase effect (*F*(1, 25) = 3.61, *p* = 0.0692, partial η^2^ = 0.13). There were no significant differences for the other antioxidant and redox-related genes (*Nfe2l2* and *Gpx1*) or for the inflammation-related gene *Tnf*. Additionally, *Akt1* and *Bmal1* exhibited phase-dependent expression, with higher levels at ZT15 (Figure S2); both effects remained significant after FDR correction (Table S2). Overall, SOL *Hmox1* expression was higher at ZT15 and tended to be higher with exercise, whereas no significant effects were detected for the other antioxidant, redox-related, and inflammation-related genes.

**Figure 4:**
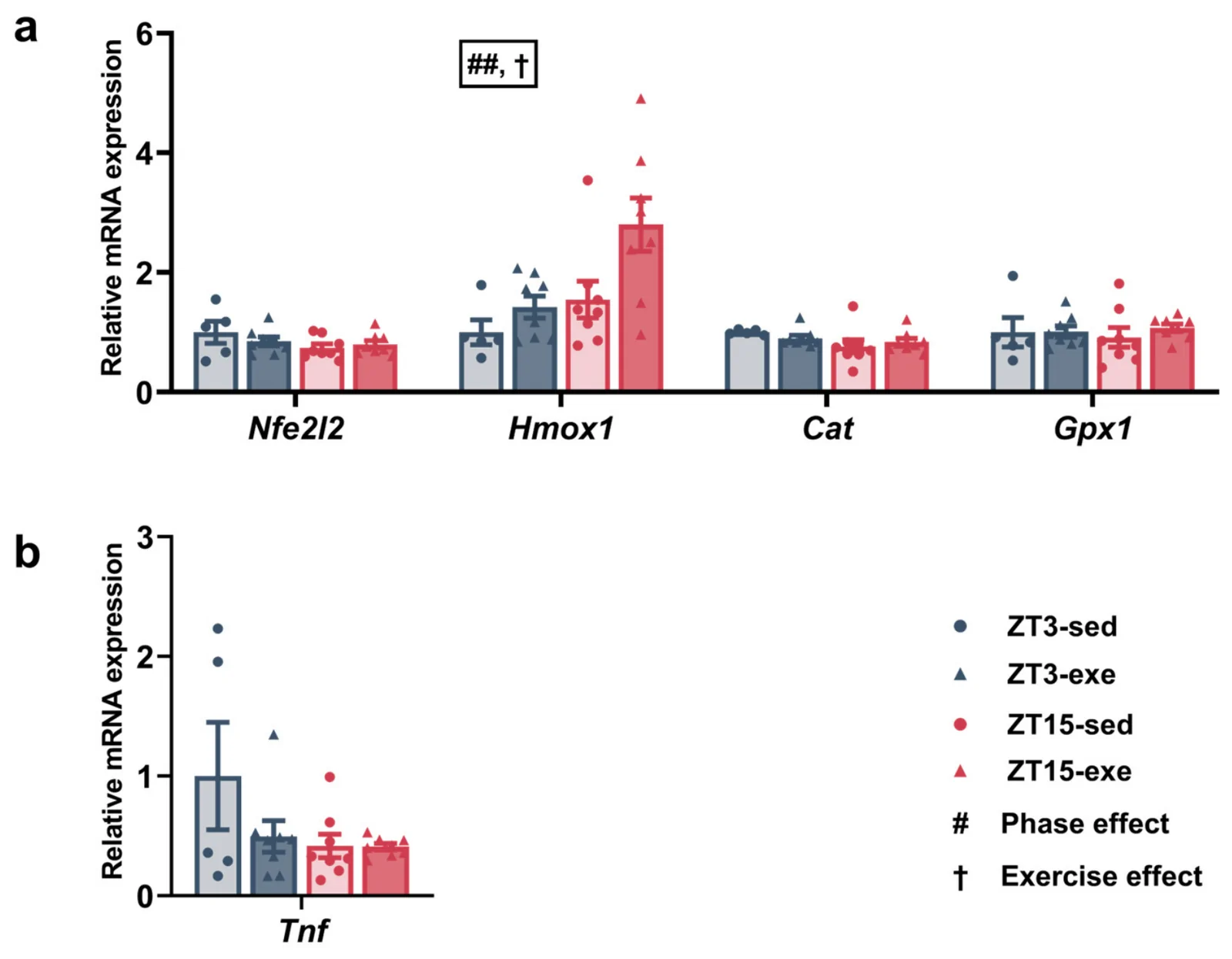
Soleus (SOL) antioxidant and inflammatory gene expression. (**a**) Antioxidant genes (*Nfe2l2*, *Hmox1, Cat,* and *Gpx1*); and (**b**) inflammatory gene (*Tnf*). Group differences were examined using two-way ANOVA with phase and exercise as factors. Data are presented as means ± SEM (ZT3-sed *n* = 5, ZT3-exe *n* = 8, ZT15-sed *n* = 8, ZT15-exe *n* = 8). # indicates a phase main effect, and † indicates an exercise main effect; † *p* < 0.05; ## *p* < 0.01. Symbols indicate unadjusted *p* values; FDR-adjusted *q* values are provided in Table S2.

### 3.4. QUA Antioxidant and Inflammatory Gene Expression (Figure 5)

In the QUA muscle, *Nfe2l2* was significantly affected by phase (*F*(1, 25) = 6.95, *p* = 0.0142, partial η^2^ = 0.22), exhibiting greater expression at ZT3 than at ZT15. In contrast, *Cat* showed a significant phase-related effect (*F*(1, 25) = 4.34, *p* = 0.0476, partial η^2^ = 0.15), with greater expression at ZT15. Neither phase effect remained significant after FDR correction (*Nfe2l2*, *q* = 0.11; *Cat*, *q* = 0.19; Table S2). The phase × exercise interaction approached significance for *Sod1* (*F*(1, 25) = 3.65, *p* = 0.0677, partial η^2^ = 0.13) and *Il1b* (*F*(1, 25) = 4.12, *p* = 0.0531, partial η^2^ = 0.14); however, exploratory *post hoc* comparisons revealed no significant pairwise differences (all *p* > 0.05). There were no significant differences for the other antioxidant and redox-related gene (*Gpx1*) or for the remaining inflammation-related genes (*Tnf*, *Il6*, and *Il10*). Overall, *Nfe2l2* and *Cat* exhibited opposite phase-dependent expression patterns in the QUA, whereas no significant exercise effects or phase × exercise interactions were detected.

**Figure 5:**
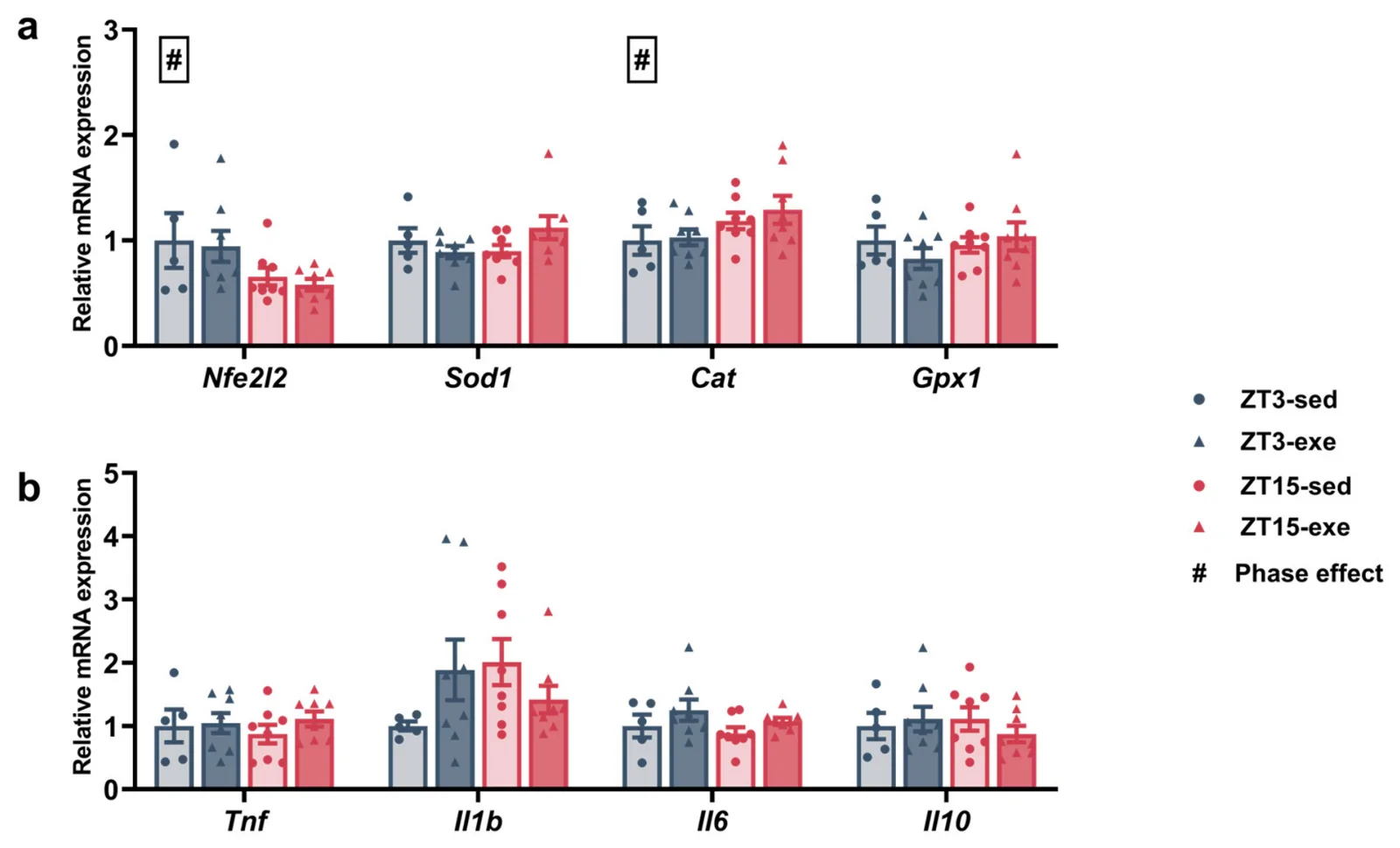
Quadriceps (QUA) antioxidant and inflammatory gene expression. (**a**) Antioxidant genes (*Nfe2l2*, *Sod1*, *Cat*, and *Gpx1*); and (**b**) inflammatory genes (*Tnf*, *Il1b*, *Il6*, and *Il10*). Group differences were examined using two-way ANOVA with phase and exercise as factors. Data are presented as means ± SEM (ZT3-sed *n* = 5, ZT3-exe *n* = 8, ZT15-sed *n* = 8, ZT15-exe *n* = 8). # indicates a phase main effect; # *p* < 0.05. Symbols indicate unadjusted *p* values; FDR-adjusted *q* values are provided in Table S2.

### 3.5. Hepatic Redox and Inflammatory Gene Expression (Figure 6)

To examine the effects of circadian phase and exercise on hepatic redox regulation and inflammation, the mRNA expression levels of *Nfe2l2*, *Keap1*, *Tnf*, *Il1b*, *Il6*, *Ccl2*, and *Adgre1* were quantified. *Keap1* exhibited a significant main effect of phase (*F*(1, 25) = 18.75, *p* = 0.0002, partial η^2^ = 0.43) and a significant interaction between phase and exercise (*F*(1, 25) = 4.97, *p* = 0.0350, partial η^2^ = 0.17), whereas the main effect of exercise was not significant. After FDR correction, the phase effect remained significant (*q* = 0.0015), whereas the interaction did not (*q* = 0.25; Table S2). *Post hoc* analysis revealed significantly greater *Keap1* expression in ZT15-sed mice than in ZT3-sed mice (mean difference = 0.61, 95% CI [0.32, 0.89], *p* = 0.0002). The phase × exercise interaction for *Adgre1* approached significance (*F*(1, 25) = 3.58, *p* = 0.0700, partial η^2^ = 0.13), but exploratory *post hoc* comparisons revealed no significant pairwise differences (all *p* > 0.05). No significant main effects or interactions were detected for *Nfe2l2* or the inflammatory genes (*Tnf*, *Il1b*, *Il6*, and *Ccl2*). In summary, hepatic *Keap1* expression was higher at ZT15 than at ZT3, particularly in sedentary mice.

**Figure 6:**
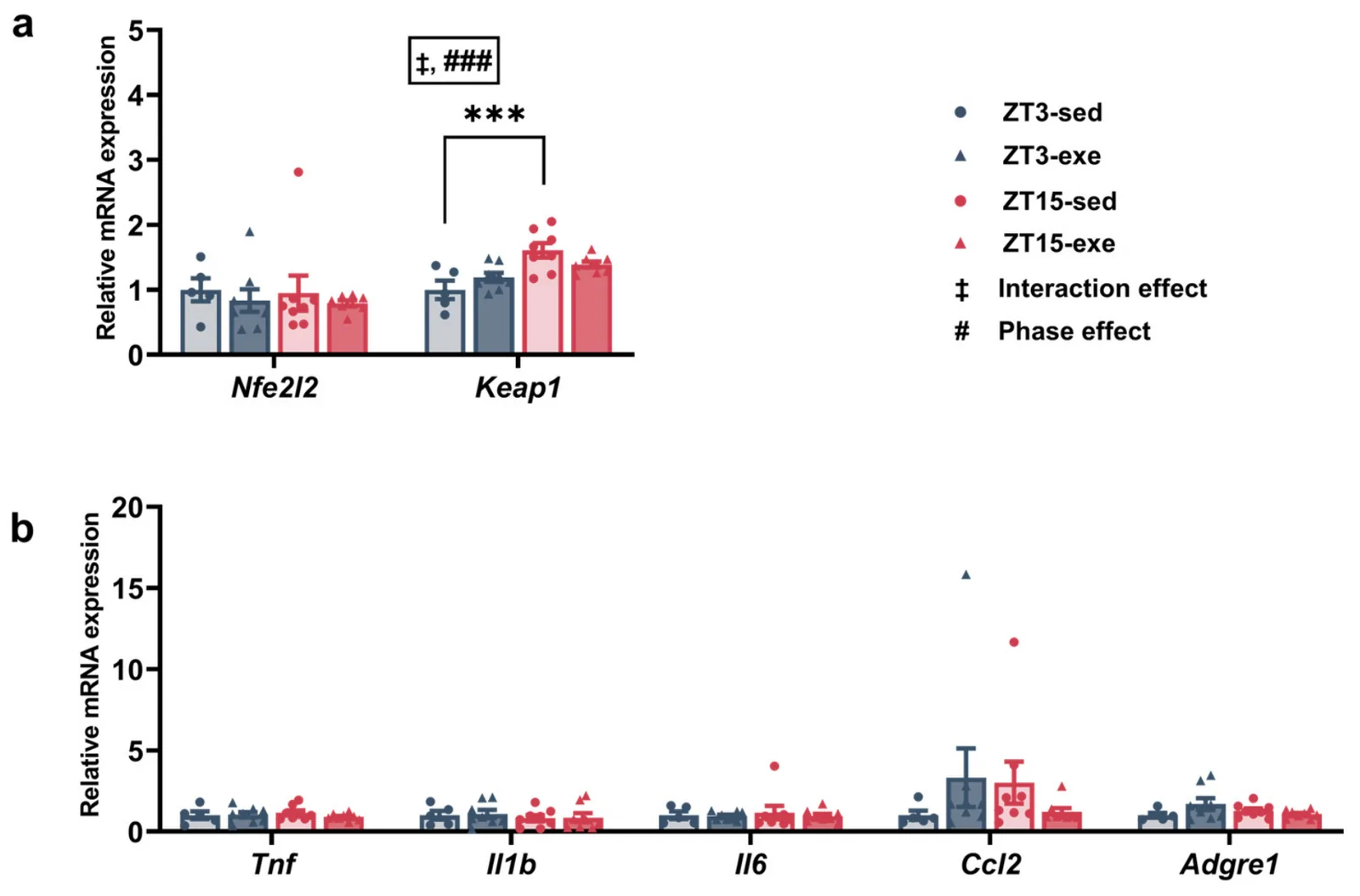
Hepatic redox and inflammatory gene expression. (**a**) Redox-related genes (*Nfe2l2* and *Keap1*); and (**b**) inflammatory genes (*Tnf*, *Il1b*, *Il6*, *Ccl2*, and *Adgre1*). Group differences were examined using two-way ANOVA with phase and exercise as factors. Data are presented as means ± SEM (ZT3-sed *n* = 5, ZT3-exe *n* = 8, ZT15-sed *n* = 8, ZT15-exe *n* = 8). ‡ indicates a phase × exercise interaction, # indicates a phase main effect, and * indicates a *post hoc* comparison; ‡ *p* < 0.05; ###/*** *p* < 0.001. Symbols indicate unadjusted *p* values; FDR-adjusted *q* values are provided in Table S2.

## 4. Discussion

### 4.1. Main Findings

The present study examined whether the circadian phase at which long-term exercise was performed influenced systemic and tissue-specific redox responses in HFD-fed mice. Overall, circadian phase, rather than a phase-specific response to exercise, was the main factor associated with tissue redox-related transcription. At the systemic level, exercise was associated with higher BAP-assessed plasma ferric-reducing capacity regardless of phase, without a parallel change in OXY-assessed HClO-neutralizing capacity, while circulating hydroperoxides showed only a possible phase-contingent pattern. At the tissue level, phase-related differences in redox-related genes were observed in all three skeletal muscles and the liver, with tissue-dependent patterns that were most robust for SOL *Hmox1* and hepatic *Keap1*, whereas inflammatory transcripts remained largely unchanged. Thus, the present data provide limited evidence that the redox response to exercise itself differed between ZT3 and ZT15.

### 4.2. Systemic Oxidative Stress and Antioxidant Responses

The systemic results suggest a distinction between a general antioxidant response to training and a more phase-dependent pattern of oxidative burden. Exercise was associated with higher BAP-assessed plasma ferric-reducing capacity, irrespective of circadian phase. Regular exercise reinforces endogenous antioxidant defenses [1], and repeated exposure to moderate exercise-induced ROS is considered an important stimulus for redox adaptation [49]. In skeletal muscle, these signals contribute to endurance adaptations [16] and fit the concept of oxidative eustress [17]. Experimental suppression of exercise-derived ROS can blunt beneficial training adaptations [18]. Thus, the higher BAP after 8 weeks of training may reflect increased systemic reducing capacity developed through repeated exercise. NRF2-mediated antioxidant signaling is one plausible contributor [19], although BAP represents the combined reducing potential of circulating components rather than the activity of a single pathway.

Plasma d-ROMs values displayed a phase × exercise interaction that approached significance. Specifically, d-ROMs were elevated in the ZT15-sed group but were lower in the ZT15-exe group at this phase, while the ZT3 groups maintained lower levels regardless of exercise status. Unlike BAP, circulating hydroperoxide accumulation appeared more sensitive to the temporal context of exercise. The lower d-ROMs value in ZT15-exe than in ZT15-sed is compatible with attenuation of the higher oxidative burden observed during the active phase under sedentary conditions. Because the interaction narrowly missed the conventional significance threshold and the pairwise comparisons were exploratory, this finding suggests a phase-contingent pattern rather than evidence that ZT15 exercise is categorically superior. Nevertheless, it agrees with evidence that exercise responses can vary by time of day [2]. Meanwhile, OXY, which reflects the capacity to neutralize specific oxidants such as HClO, showed no significant differences among groups. The different responses of OXY, BAP, and d-ROMs are not necessarily contradictory because the assays quantify different aspects of circulating redox status: HClO-buffering capacity, ferric-reducing potential, and hydroperoxide load, respectively. Circulating antioxidant-capacity assays can also differ in their sensitivity to individual plasma constituents [50], and assays based on different principles show limited agreement with one another, supporting the combined use of complementary assays [51]. Taken together, training was associated with higher plasma ferric-reducing capacity, whereas d-ROMs showed a possible phase-contingent pattern.

### 4.3. Tissue- and Muscle-Specific Transcriptional Adaptations

Phase-related differences in selected redox-related transcripts were observed in all three muscles, but their patterns differed among muscles with different fiber-type compositions. This divergence may reflect the “spatially discrete signaling” of ROS within different muscle environments, where mitochondrial quality control and adaptive responses are tailored to the specific energetic demands of the tissue [20].

In the oxidative SOL, *Hmox1* showed both a phase main effect (higher at ZT15) and an exercise main effect (higher with training), of which the phase effect was more robust. This pattern is consistent with a possible training-related cytoprotective response superimposed on phase-dependent expression. HO-1 is induced by redox-sensitive stress signaling, and a training-related increase is consistent with adaptation to repeated ROS exposure. Exercise-responsive ROS pathways contribute to antioxidant regulation and mitochondrial adaptation in skeletal muscle [52]. GAS and QUA showed different patterns from SOL. In GAS, the *Nfe2l2* interaction appears to reflect modification of an underlying phase difference rather than a uniform exercise-induced increase, because the pairwise difference was observed between the sedentary groups. In QUA, higher *Nfe2l2* at ZT3 and higher *Cat* at ZT15 suggest that phase may affect antioxidant components in opposite directions without a detectable training effect. The supplementary clock-gene results were consistent with distinct temporal states in skeletal muscle, in line with evidence that scheduled exercise can influence the muscle molecular clock [13]. Consistent with the phase-sensitive QUA response, our previous study of QUA-derived extracellular vesicle (EV)-enriched fractions showed that circadian phase contributed more than exercise to the overall variation in EV microRNA (miRNA) profiles. Core target genes derived from miRNAs that were higher in the composite ZT15-versus-ZT3 contrast were enriched in mitochondrion organization and positive regulation of reactive oxygen species biosynthetic process, whereas those derived from miRNAs that were lower in this contrast were enriched in heme biosynthetic process and intracellular iron ion homeostasis [53]. This suggests that phase-related redox regulation in QUA may also be reflected in EV-associated miRNA profiles.

These patterns agree with evidence that antioxidant enzyme responses vary with muscle fiber composition [22]. Differences in mitochondrial content, substrate use, and ROS sources between the oxidative SOL and the more glycolytic or mixed GAS and QUA may therefore contribute to the observed responses. This heterogeneity is consistent with a muscle-specific redox landscape [23]. Collectively, these findings are compatible with the view that muscle metabolic phenotype and circadian phase jointly shape redox-related transcriptional responses. The most robust muscle response was the phase-dependent expression of SOL *Hmox1*, whereas the exercise effect in SOL and the phase-related patterns in GAS and QUA require confirmation in larger cohorts.

### 4.4. Hepatic Redox and Metabolic Gene Regulation

In the liver, among the antioxidant-related genes examined, only *Keap1* showed significant phase-dependent modulation, with higher expression at ZT15 that was most evident under sedentary conditions; hepatic *Nfe2l2* itself did not differ significantly across groups in the present dataset. This partially aligns with prior work reporting circadian variation in hepatic antioxidant gene expression [15]. Because Kelch-like ECH-associated protein 1 (KEAP1) regulates the stability, degradation, and cellular localization of NRF2 [54], the higher *Keap1* mRNA level at ZT15 suggests a phase-dependent difference in the regulatory environment of NRF2 signaling. The nominal phase × exercise interaction further suggests that this phase difference in sedentary mice may be altered by training, even though exercise did not produce a uniform main effect on hepatic *Keap1*.

The liver is strongly organized by circadian and metabolic inputs. Its molecular clock coordinates daily metabolic functions [55], and feeding-related signals help maintain hepatic metabolic rhythms [56]. The hepatic clock and NRF2–HO-1 system can also undergo redox-related reprogramming in fatty liver [29]. Our previous study showed that hepatic triglyceride content was lower at ZT3 and *Cpt1a* expression was higher at ZT3 and under sedentary conditions, whereas *Ppargc1a*, *Slc27a2*, and *Slc27a5* expression did not differ significantly by phase. Exercise reduced the Oil Red O-positive area without a coordinated increase in these genes [28]. Thus, the phase-sensitive *Keap1* response occurred within a temporally organized hepatic metabolic environment, although the lipid and redox outcomes did not change in parallel at the mRNA level. This interpretation is consistent with tissue-specific coupling between circadian metabolism and redox regulation [30].

### 4.5. Inflammatory Responses and EV miRNA Context

The redox-related differences were not accompanied by persistent changes in the inflammatory transcripts measured at 48 h after the last exercise bout. This pattern contrasts with reports that training suppresses HFD-induced inflammation in other organs, including the lung [40], possibly because of differences in tissue and sampling time. Circadian-redox signaling can regulate inflammatory output in immune cells; BMAL1, for example, regulates macrophage interleukin-1β production through NRF2 [57]. Here, the stable muscle and liver cytokine transcripts indicate that the observed redox responses occurred without a broad alteration of local inflammatory gene expression.

Our previous EV-miRNA study also showed that core target genes derived from miRNAs that were higher in the composite exercise-versus-sedentary contrast were enriched in negative regulation of canonical nuclear factor kappa B (NF-κB) signal transduction, whereas those derived from miRNAs that were lower in this contrast were enriched in positive regulation of interleukin-1 beta production [53]. Kyoto Encyclopedia of Genes and Genomes (KEGG) analysis identified the TNF signaling pathway in the former target set and the Toll-like receptor signaling pathway in the latter [53]. Thus, although the present study detected no persistent changes in local cytokine transcripts, inflammatory processes were represented in the predicted EV-miRNA target networks. However, enrichment analysis does not establish pathway activation or suppression, and the composite exercise-versus-sedentary contrast may partly reflect phase-dependent miRNA differences.

Together, these findings suggest that circadian phase may influence how redox responses are distributed across biological compartments rather than simply determining whether exercise is beneficial. This agrees with the literature identifying exercise timing as a modifier of metabolic adaptation [2]. Exercise can also act as a timing cue for peripheral tissues [58], providing a framework for the phase-sensitive responses observed here.

### 4.6. Tryptophan–Melatonin Pathway and Melatonin-Independent Timing Cues

Because circadian phase was the main factor associated with the tissue responses observed here, it is relevant to consider which timing signals may convey phase information to peripheral tissues. Melatonin, synthesized from tryptophan via serotonin, is a major hormonal output of the circadian system and acts both as a free-radical scavenger and as an inducer of antioxidant enzymes [59]; in fatty liver, it has been reported to restore NRF2–HO-1 signaling and hepatic clock oscillations [29]. However, C57BL/6J mice carry genetic defects in melatonin-synthesizing enzymes and produce little or no pineal melatonin [60,61], and melatonin productivity has no apparent effect on circadian behavior in mice [61]. The phase-related differences in *Hmox1*, *Nfe2l2*, *Cat*, and *Keap1* observed here, particularly those in SOL *Hmox1* and hepatic *Keap1*, are therefore more likely to reflect melatonin-independent mechanisms, such as peripheral tissue clocks entrained by feeding and exercise [56,58], than nocturnal melatonin signaling. Whether melatonin and other tryptophan metabolites modify phase-dependent redox responses to exercise remains to be examined in melatonin-proficient models or in humans.

### 4.7. Limitations

Several limitations warrant consideration. First, the small sample sizes and unequal group sizes (*n* = 5–8) limited power to detect subtle interactions, and multiple outcomes were analyzed without a prespecified primary endpoint; apart from the phase effects on SOL *Hmox1* and hepatic *Keap1* and the supplementary genes, the observed effects did not withstand FDR correction and should be regarded as hypothesis-generating. Second, ZT3 and ZT15 provide a two-phase contrast rather than a complete 24-h rhythm, and samples were collected at a single time point. Third, tissue outcomes were restricted to mRNA levels in bulk tissue; protein abundance, enzyme activity, and markers of oxidative damage were not measured, and hepatocyte ploidy, which can be altered by oxidative stress in fatty liver, was not assessed [62]. Fourth, only the ZT15 group underwent constant light and light–dark reversal, and exercise at ZT3 was performed during the rest phase, when sleep may have been disturbed; sleep was not monitored. Fifth, extrapolation to non-obese animals, female animals, or human populations should be approached with caution.

## 5. Conclusions

This study examined the effects of circadian phase and long-term exercise training on systemic and tissue-specific redox responses under HFD conditions. Exercise was associated with higher plasma ferric-reducing capacity irrespective of circadian phase, whereas OXY-assessed HClO-neutralizing capacity was unchanged. Circulating hydroperoxides (d-ROMs) showed only a trend toward a phase × exercise interaction, with exploratory comparisons tentatively suggesting higher levels in ZT15-sed mice. At the molecular level, SOL *Hmox1* expression was higher at ZT15 and, less robustly, in exercised mice, whereas *Nfe2l2* and *Cat* in QUA differed by phase in opposite directions without detectable training effects. In GAS (*Nfe2l2*) and the liver (*Keap1*), expression was higher in ZT15-sed than in ZT3-sed mice, a difference not evident among exercised mice. Inflammatory transcripts in skeletal muscle and liver remained largely unchanged. Together, these exploratory findings suggest that long-term exercise was associated with higher plasma ferric-reducing capacity, whereas tissue transcriptional responses were mainly associated with circadian phase and differed among tissues. Whether active-phase exercise produces more favorable redox adaptations than rest-phase exercise requires further investigation using additional circadian time points, protein- and functional-level measurements, and female animals.

## Figures and Tables

**Table 1:** Primer sequences used for quantitative real-time polymerase chain reaction (qPCR) analysis.

Gene	Forward	Reverse
*Adgre1*	CAACCTGCCACAACACTCTC	GCACTCATCCACATCTTCACA
*Cat*	GATGGAGAGGCAGTCTATTG	ATTGGCGATGGCATTGAA
*Ccl2*	GCATCTGCCCTAAGGTCTTCA	TCACTGTCACACTGGTCACTC
*Gapdh*	TCTCCTGCGACTTCAACA	TGTAGCCGTATTCATTGTCA
*Gpx1*	CAAGGTGCTGCTCATTGA	TGGTGTCCGAACTGATTG
*Hmox1*	GCCTTCCTGCTCAACATT	AAGTGACGCCATCTGTGA
*Il10*	ACATACTGCTAACCGACTCCT	GGCATCACTTCTACCAGGTAA
*Il1b*	CACTACAGGCTCCGAGATGA	CGTTGCTTGGTTCTCCTTGT
*Il6*	CTTGGGACTGATGCTGGTGACA	GCCTCCGACTTGTGAAGTGGTA
*Keap1*	ATGTTGACACGGAGGATTG	ACATTCTGCGGAGTTAAGC
*Nfe2l2*	GCCTCTAGTGAGAAGAACAA	GTGACCAACTGAACGATAAC
*Rps18*	TTCTGGCCAACGGTCTAGACAAC	CCAGTGGTCTTGGTGTGCTGA
*Sod1*	AGTTGCCACCGCCAGGACTA	CGTGCTCAGAAACCTCCTTCCAAA
*Sod2*	GAACCAGTTGTGTTGTCAG	GTACAGCCTTGTGTATTGTC
*Tnf*	CAACTCAGGTCGCTCTTC	TGATAGCCTCCAGCAACT

Forward and reverse primer sequences are presented in the 5′-to-3′ direction. The target genes comprised redox-regulatory and antioxidant genes: *Nfe2l2* (nuclear factor, erythroid derived 2, like 2), *Keap1* (kelch-like ECH-associated protein 1), *Hmox1* (heme oxygenase 1), *Sod1* (superoxide dismutase 1), *Sod2* (superoxide dismutase 2), *Cat* (catalase), and *Gpx1* (glutathione peroxidase 1); and inflammatory and immune genes: *Tnf* (tumor necrosis factor), *Il1b* (interleukin 1 beta), *Il6* (interleukin 6), *Il10* (interleukin 10), *Ccl2* (C-C motif chemokine ligand 2), and *Adgre1* (adhesion G protein-coupled receptor E1). *Gapdh* (glyceraldehyde-3-phosphate dehydrogenase) was used as the reference gene for gastrocnemius (GAS), whereas *Rps18* (ribosomal protein S18) was used for soleus (SOL), quadriceps (QUA), and liver.

## Data Availability

The original contributions presented in this study are included in the article and Supplementary Materials. Further inquiries can be directed to the corresponding author.

## Supplementary Materials

The following supporting information can be downloaded at: https://www.mdpi.com/article/10.3390/nu18203384/s1, Table S1: Primer sequences used for supplementary quantitative real-time polymerase chain reaction (qPCR) analyses; Table S2: Two-way ANOVA results with FDR-adjusted *q* values; Figure S1: Core clock gene expression in GAS; Figure S2: *Bmal1* and *Akt1* expression in SOL.

## Abbreviations

The following abbreviations are used in this manuscript:

ANOVA	Analysis of variance
ARRIVE	Animal Research: Reporting of In Vivo Experiments
BAP	Biological antioxidant potential test
BMAL1	Brain and muscle ARNT-like 1
CARR U	Carratelli units
CAT	Catalase
cDNA	Complementary DNA
CI	Confidence interval
CLOCK	Circadian locomotor output cycles kaput
Ct	Cycle threshold
DEPPD	*N,N*-diethyl-*p*-phenylenediamine
d-ROMs	Derivatives of reactive oxygen metabolites test
EV	Extracellular vesicle
exe	Exercise
FDR	False discovery rate
FREE	Free Radical Elective Evaluator
GAS	Gastrocnemius
GPX1	Glutathione peroxidase 1
HClO	Hypochlorous acid
HFD	High-fat diet
HO-1	Heme oxygenase-1
KEAP1	Kelch-like ECH-associated protein 1
KEGG	Kyoto Encyclopedia of Genes and Genomes
miRNA	microRNA
mRNA	Messenger RNA
NF-κB	Nuclear factor kappa B
NQO1	NAD(P)H:quinone oxidoreductase 1
NRF2	Nuclear factor erythroid 2-related factor 2
OXY	OXY-adsorbent test
qPCR	Quantitative real-time polymerase chain reaction
QUA	Quadriceps
ROS	Reactive oxygen species
sed	Sedentary
SEM	Standard error of the mean
SOL	Soleus
SOD2	Superoxide dismutase 2
VO_2_max	Maximal oxygen uptake
ZT	Zeitgeber time

## References

[1] Gomez-Cabrera M.-C., Domenech E., Viña J. (2008). Moderate Exercise Is an Antioxidant: Upregulation of Antioxidant Genes by Training. Free Radic. Biol. Med..

[2] Sato S., Basse A.L., Schönke M., Chen S., Samad M., Altıntaş A., Laker R.C., Dalbram E., Barrès R., Baldi P. (2019). Time of Exercise Specifies the Impact on Muscle Metabolic Pathways and Systemic Energy Homeostasis. Cell Metab..

[3] Ezagouri S., Zwighaft Z., Sobel J., Baillieul S., Doutreleau S., Ladeuix B., Golik M., Verges S., Asher G. (2019). Physiological and Molecular Dissection of Daily Variance in Exercise Capacity. Cell Metab..

[4] Yamazaki S., Numano R., Abe M., Hida A., Takahashi R., Ueda M., Block G.D., Sakaki Y., Menaker M., Tei H. (2000). Resetting Central and Peripheral Circadian Oscillators in Transgenic Rats. Science.

[5] Yoo S.-H., Yamazaki S., Lowrey P.L., Shimomura K., Ko C.H., Buhr E.D., Siepka S.M., Hong H.-K., Oh W.J., Yoo O.J. (2004). PERIOD2::LUCIFERASE Real-Time Reporting of Circadian Dynamics Reveals Persistent Circadian Oscillations in Mouse Peripheral Tissues. Proc. Natl. Acad. Sci. USA.

[6] Bass J., Takahashi J.S. (2010). Circadian Integration of Metabolism and Energetics. Science.

[7] Partch C.L., Green C.B., Takahashi J.S. (2014). Molecular Architecture of the Mammalian Circadian Clock. Trends Cell Biol..

[8] Gekakis N., Staknis D., Nguyen H.B., Davis F.C., Wilsbacher L.D., King D.P., Takahashi J.S., Weitz C.J. (1998). Role of the CLOCK Protein in the Mammalian Circadian Mechanism. Science.

[9] Preitner N., Damiola F., Lopez-Molina L., Zakany J., Duboule D., Albrecht U., Schibler U. (2002). The Orphan Nuclear Receptor REV-ERBα Controls Circadian Transcription within the Positive Limb of the Mammalian Circadian Oscillator. Cell.

[10] Turek F.W., Joshu C., Kohsaka A., Lin E., Ivanova G., McDearmon E., Laposky A., Losee-Olson S., Easton A., Jensen D.R. (2005). Obesity and Metabolic Syndrome in Circadian *Clock* Mutant Mice. Science.

[11] Gutierrez-Monreal M.A., Wolff C.A., Rijos E.E., Viggars M.R., Douglas C.M., Pagala V., Peng J., Hunt L.C., Ding H., Huo Z. (2024). Targeted Bmal1 Restoration in Muscle Prolongs Lifespan with Systemic Health Effects in Aging Model. JCI Insight.

[12] Andrews J.L., Zhang X., McCarthy J.J., McDearmon E.L., Hornberger T.A., Russell B., Campbell K.S., Arbogast S., Reid M.B., Walker J.R. (2010). CLOCK and BMAL1 Regulate *MyoD* and Are Necessary for Maintenance of Skeletal Muscle Phenotype and Function. Proc. Natl. Acad. Sci. USA.

[13] Wolff G., Esser K.A. (2012). Scheduled Exercise Phase Shifts the Circadian Clock in Skeletal Muscle. Med. Sci. Sports Exerc..

[14] Pekovic-Vaughan V., Gibbs J., Yoshitane H., Yang N., Pathiranage D., Guo B., Sagami A., Taguchi K., Bechtold D., Loudon A. (2014). The Circadian Clock Regulates Rhythmic Activation of the NRF2/Glutathione-Mediated Antioxidant Defense Pathway to Modulate Pulmonary Fibrosis. Genes Dev..

[15] Xu Y.-Q., Zhang D., Jin T., Cai D.-J., Wu Q., Lu Y., Liu J., Klaassen C.D. (2012). Diurnal Variation of Hepatic Antioxidant Gene Expression in Mice. PLoS ONE.

[16] Powers S.K., Radak Z., Ji L.L., Jackson M. (2024). Reactive Oxygen Species Promote Endurance Exercise-Induced Adaptations in Skeletal Muscles. J. Sport Health Sci..

[17] Sies H. (2021). Oxidative Eustress: On Constant Alert for Redox Homeostasis. Redox Biol..

[18] Gomez-Cabrera M.-C., Borrás C., Pallardó F.V., Sastre J., Ji L.L., Viña J. (2005). Decreasing Xanthine Oxidase-Mediated Oxidative Stress Prevents Useful Cellular Adaptations to Exercise in Rats. J. Physiol..

[19] Vargas-Mendoza N., Morales-González Á., Madrigal-Santillán E.O., Madrigal-Bujaidar E., Álvarez-González I., García-Melo L.F., Anguiano-Robledo L., Fregoso-Aguilar T., Morales-Gonzalez J.A. (2019). Antioxidant and Adaptative Response Mediated by Nrf2 during Physical Exercise. Antioxidants.

[20] Craige S.M., Mammel R.K., Amiri N., Willoughby O.S., Drake J.C. (2024). Interplay of ROS, Mitochondrial Quality, and Exercise in Aging: Potential Role of Spatially Discrete Signaling. Redox Biol..

[21] Jyothidasan A., Sunny S., Devarajan A., Sayed A., Afortude J.K., Dalley B., Nanda V., Pogwizd S., Litovsky S.H., Trinity J.D. (2024). Exercise Mitigates Reductive Stress-Induced Cardiac Remodeling in Mice. Redox Biol..

[22] Powers S.K., Criswell D., Lawler J., Ji L.L., Martin D., Herb R.A., Dudley G. (1994). Influence of Exercise and Fiber Type on Antioxidant Enzyme Activity in Rat Skeletal Muscle. Am. J. Physiol..

[23] Sutton E., Pekovic-Vaughan V. (2025). Time to Reset: The Interplay Between Circadian Rhythms and Redox Homeostasis in Skeletal Muscle Ageing and Systemic Health. Antioxidants.

[24] Shimba S., Ishii N., Ohta Y., Ohno T., Watabe Y., Hayashi M., Wada T., Aoyagi T., Tezuka M. (2005). Brain and Muscle Arnt-like Protein-1 (BMAL1), a Component of the Molecular Clock, Regulates Adipogenesis. Proc. Natl. Acad. Sci. USA.

[25] Bugge A., Feng D., Everett L.J., Briggs E.R., Mullican S.E., Wang F., Jager J., Lazar M.A. (2012). Rev-Erbα and Rev-Erbβ Coordinately Protect the Circadian Clock and Normal Metabolic Function. Genes Dev..

[26] Dyar K.A., Ciciliot S., Wright L.E., Biensø R.S., Tagliazucchi G.M., Patel V.R., Forcato M., Paz M.I.P., Gudiksen A., Solagna F. (2014). Muscle Insulin Sensitivity and Glucose Metabolism Are Controlled by the Intrinsic Muscle Clock. Mol. Metab..

[27] Kohsaka A., Laposky A.D., Ramsey K.M., Estrada C., Joshu C., Kobayashi Y., Turek F.W., Bass J. (2007). High-Fat Diet Disrupts Behavioral and Molecular Circadian Rhythms in Mice. Cell Metab..

[28] Wang S., Zhang Z., Huang J., Tong Y., Wu C., Kobori H., Ma S., Suzuki K. (2025). Circadian Phase Determines Tissue-Specific Adaptations to Long-Term Exercise in Obese Mice. Nutrients.

[29] Joshi A., Upadhyay K.K., Vohra A., Shirsath K., Devkar R. (2021). Melatonin Induces Nrf2-HO-1 Reprogramming and Corrections in Hepatic Core Clock Oscillations in Non-alcoholic Fatty Liver Disease. FASEB J..

[30] Konakchieva R., Mladenov M., Konaktchieva M., Sazdova I., Gagov H., Nikolaev G. (2025). Circadian Clock Deregulation and Metabolic Reprogramming: A System Biology Approach to Tissue-Specific Redox Signaling and Disease Development. Int. J. Mol. Sci..

[31] Pendergrast L.A., Lundell L.S., Ehrlich A.M., Ashcroft S.P., Schönke M., Basse A.L., Krook A., Treebak J.T., Dollet L., Zierath J.R. (2023). Time of Day Determines Postexercise Metabolism in Mouse Adipose Tissue. Proc. Natl. Acad. Sci. USA.

[32] Nelson R.J., DeVries A.C., Prendergast B.J. (2024). Researchers Need to Better Address Time-of-Day as a Critical Biological Variable. Proc. Natl. Acad. Sci. USA.

[33] Casanova-Vallve N., Duglan D., Vaughan M.E., Pariollaud M., Handzlik M.K., Fan W., Yu R.T., Liddle C., Downes M., Delezie J. (2022). Daily Running Enhances Molecular and Physiological Circadian Rhythms in Skeletal Muscle. Mol. Metab..

[34] Adamovich Y., Dandavate V., Ezagouri S., Manella G., Zwighaft Z., Sobel J., Kuperman Y., Golik M., Auerbach A., Itkin M. (2021). Clock Proteins and Training Modify Exercise Capacity in a Daytime-Dependent Manner. Proc. Natl. Acad. Sci. USA.

[35] Kovynev A., Ying Z., Zhang S., Olgiati E., Lambooij J.M., Visentin C., Guigas B., Ducarmon Q.R., Rensen P.C.N., Schönke M. (2024). Timing Matters: Late, but Not Early, Exercise Training Ameliorates MASLD in Part by Modulating the Gut-Liver Axis in Mice. J. Pineal Res..

[36] Yamanaka Y., Hashimoto S., Takasu N.N., Tanahashi Y., Nishide S., Honma S., Honma K. (2015). Morning and Evening Physical Exercise Differentially Regulate the Autonomic Nervous System during Nocturnal Sleep in Humans. Am. J. Physiol.-Regul. Integr. Comp. Physiol..

[37] Moholdt T., Parr E.B., Devlin B.L., Debik J., Giskeødegård G., Hawley J.A. (2021). The Effect of Morning vs Evening Exercise Training on Glycaemic Control and Serum Metabolites in Overweight/Obese Men: A Randomised Trial. Diabetologia.

[38] Lynch H.J., Jimerson D.C., Ozaki Y., Post R.M., Bunney W.E., Wurtman R.J. (1978). Entrainment of Rhythmic Melatonin Secretion in Man to a 12-Hour Phase Shift in the Light/Dark Cycle. Life Sci..

[39] Sudo A., Miki K. (1995). Circadian Rhythm of Catecholamine Excretion in Rats after Phase Shift of Light-Dark Cycle. Ind. Health.

[40] Cho J., Johnson B.D., Watt K.D., Niven A.S., Yeo D., Kim C.-H. (2022). Exercise Training Attenuates Pulmonary Inflammation and Mitochondrial Dysfunction in a Mouse Model of High-Fat High-Carbohydrate-Induced NAFLD. BMC Med..

[41] Xu Z., Qin Y., Lv B., Tian Z., Zhang B. (2022). Effects of Moderate-Intensity Continuous Training and High-Intensity Interval Training on Testicular Oxidative Stress, Apoptosis and m6A Methylation in Obese Male Mice. Antioxidants.

[42] Vassalle C. (2008). An Easy and Reliable Automated Method to Estimate Oxidative Stress in the Clinical Setting. Methods Mol. Biol..

[43] Tong Y., Ma S., Awa R., Tagawa T., Seki Y., Cao T., Kobori H., Suzuki K. (2025). Effects of 3-(4-Hydroxy-3-Methoxyphenyl)Propionic Acid on Regulating Oxidative Stress and Muscle Fiber Composition. Nutrients.

[44] Katerji M., Filippova M., Duerksen-Hughes P. (2019). Approaches and Methods to Measure Oxidative Stress in Clinical Samples: Research Applications in the Cancer Field. Oxid. Med. Cell. Longev..

[45] Trotti R., Carratelli M., Barbieri M. (2002). Performance and Clinical Application of a New, Fast Method for the Detection of Hydroperoxides in Serum. Panminerva Med..

[46] Pigazzani F., Gorni D., Dyar K.A., Pedrelli M., Kennedy G., Costantino G., Bruno A., Mackenzie I., MacDonald T.M., Tietge U.J.F. (2022). The Prognostic Value of Derivatives-Reactive Oxygen Metabolites (d-ROMs) for Cardiovascular Disease Events and Mortality: A Review. Antioxidants.

[47] Alberti A., Bolognini L., Macciantelli D., Caratelli M. (2000). The Radical Cation of N,N-Diethyl-Para-Phenylendiamine: A Possible Indicator of Oxidative Stress in Biological Samples. Res. Chem. Intermed..

[48] Costantini D. (2016). Oxidative Stress Ecology and the D-ROMs Test: Facts, Misfacts and an Appraisal of a Decade’s Work. Behav. Ecol. Sociobiol..

[49] Powers S.K., Radak Z., Ji L.L. (2016). Exercise-Induced Oxidative Stress: Past, Present and Future. J. Physiol..

[50] Costantini D. (2011). On the Measurement of Circulating Antioxidant Capacity and the Nightmare of Uric Acid. Methods Ecol. Evol..

[51] Jansen E., Ruskovska T. (2013). Comparative Analysis of Serum (Anti)Oxidative Status Parameters in Healthy Persons. Int. J. Mol. Sci..

[52] Bouviere J., Fortunato R.S., Dupuy C., Werneck-de-Castro J.P., Carvalho D.P., Louzada R.A. (2021). Exercise-Stimulated ROS Sensitive Signaling Pathways in Skeletal Muscle. Antioxidants.

[53] Wang S., Kawanishi N., Wu C., Kobori H., Suzuki K. (2026). Circadian Phase Shapes Muscle-Derived Extracellular Vesicle microRNA Profiles with Context-Dependent Modulation by Exercise in High-Fat-Diet-Fed Mice. Nutrients.

[54] Itoh K., Wakabayashi N., Katoh Y., Ishii T., O’Connor T., Yamamoto M. (2003). Keap1 Regulates Both Cytoplasmic-Nuclear Shuttling and Degradation of Nrf2 in Response to Electrophiles. Genes Cells.

[55] Reinke H., Asher G. (2016). Circadian Clock Control of Liver Metabolic Functions. Gastroenterology.

[56] Greco C.M., Koronowski K.B., Smith J.G., Shi J., Kunderfranco P., Carriero R., Chen S., Samad M., Welz P.-S., Zinna V.M. (2021). Integration of Feeding Behavior by the Liver Circadian Clock Reveals Network Dependency of Metabolic Rhythms. Sci. Adv..

[57] Early J.O., Menon D., Wyse C.A., Cervantes-Silva M.P., Zaslona Z., Carroll R.G., Palsson-McDermott E.M., Angiari S., Ryan D.G., Corcoran S.E. (2018). Circadian Clock Protein BMAL1 Regulates IL-1β in Macrophages via NRF2. Proc. Natl. Acad. Sci. USA.

[58] Gabriel B.M., Zierath J.R. (2019). Circadian Rhythms and Exercise—Re-Setting the Clock in Metabolic Disease. Nat. Rev. Endocrinol..

[59] Reiter R.J., Mayo J.C., Tan D., Sainz R.M., Alatorre-Jimenez M., Qin L. (2016). Melatonin as an Antioxidant: Under Promises but over Delivers. J. Pineal Res..

[60] Ebihara S., Marks T., Hudson D.J., Menaker M. (1986). Genetic Control of Melatonin Synthesis in the Pineal Gland of the Mouse. Science.

[61] Kasahara T., Abe K., Mekada K., Yoshiki A., Kato T. (2010). Genetic Variation of Melatonin Productivity in Laboratory Mice under Domestication. Proc. Natl. Acad. Sci. USA.

[62] Gentric G., Maillet V., Paradis V., Couton D., L’Hermitte A., Panasyuk G., Fromenty B., Celton-Morizur S., Desdouets C. (2015). Oxidative Stress Promotes Pathologic Polyploidization in Nonalcoholic Fatty Liver Disease. J. Clin. Investig..

