# Supplementary material for: Effects of Circadian Phase and Exercise Training on Systemic and Tissue-Specific Redox Responses in Mice Fed a High-Fat Diet
Source: Nutrients. 2026 Oct 15;18(20):3384. doi: 10.3390/nu18203384 (PMC13708115; doi:10.3390/nu18203384)
Supplement: Supplementary file 1 [file nutrients-18-03384-s001.zip › nutrients-4602432-supplementary.pdf]

Table S1. Primer sequences used for supplementary quantitative real-time polymerase chain reaction (qPCR) analyses.

Gene	Forward	Reverse
Bmal1	GGACTTCGCCTCTACCTGTT	CCTCGTTGTCTGGCTCATTG
Cry1	GCTGGCGTGGAAGTCATCGT	ATGGTGTCTGCTGGCATCTCC
Nr1d1	ACGGCAAGGCAACACCAA	GCGGCTCAGGAACATCACT
Per2	GCTGCGGATGCTCGTGGAAT	GGTTGTGCTCTGCCTCTGTCAT
Akt1	CCAAGCACCGTGTGACCATGAA	TGGCGACGATGACCTCCTTCTT

Forward and reverse primer sequences are presented in the 5′-to-3′ direction. The supplementary analyses included the core clock genes *Bmal1* (basic helix-loop-helix ARNT like 1), *Cry1* (cryptochrome circadian regulator 1), *Nr1d1* (nuclear receptor subfamily 1, group D, member 1), and *Per2* (period circadian clock 2) in gastrocnemius (GAS), and *Bmal1* and the signaling gene *Akt1* (Akt serine/threonine kinase 1) in soleus (SOL). *Gapdh* and *Rps18* were used as the reference genes for GAS and SOL, respectively, as described in Table 1.

Table S2. Two-way ANOVA results with false discovery rate (FDR)-adjusted q values.

**Family**	**Outcome**	**Effect**	$F(1, 25)$	p	ηp^2	q **(FDR)**
Plasma	OXY	Phase	0.35	0.560	0.01	0.840
Plasma	OXY	Exercise	0.07	0.792	0.00	0.792
Plasma	OXY	Phase × Exercise	0.25	0.624	0.01	0.624
Plasma	d-ROMs	Phase	2.17	0.154	0.08	0.461
Plasma	d-ROMs	Exercise	2.43	0.131	0.09	0.197
Plasma	d-ROMs	Phase × Exercise	4.02	0.056	0.14	0.168
Plasma	BAP	Phase	0.03	0.863	0.00	0.863
Plasma	BAP	Exercise	4.49	0.044	0.15	0.133
Plasma	BAP	Phase × Exercise	0.72	0.403	0.03	0.605
GAS	*Nfe2l2*	Phase	0.86	0.363	0.03	0.415
GAS	*Nfe2l2*	Exercise	0.01	0.928	0.00	0.928
GAS	*Nfe2l2*	Phase × Exercise	4.86	0.037	0.16	0.296
GAS	*Hmox1*	Phase	1.40	0.247	0.05	0.396
GAS	*Hmox1*	Exercise	0.26	0.612	0.01	0.841
GAS	*Hmox1*	Phase × Exercise	0.00	0.945	0.00	0.945
GAS	*Sod2*	Phase	2.71	0.112	0.10	0.396
GAS	*Sod2*	Exercise	0.24	0.631	0.01	0.841
GAS	*Sod2*	Phase × Exercise	1.48	0.235	0.06	0.568
GAS	*Cat*	Phase	1.60	0.218	0.06	0.396
GAS	*Cat*	Exercise	0.44	0.512	0.02	0.841
GAS	*Cat*	Phase × Exercise	0.01	0.935	0.00	0.945
GAS	*Gpx1*	Phase	0.88	0.358	0.03	0.415
GAS	*Gpx1*	Exercise	0.01	0.908	0.00	0.928
GAS	*Gpx1*	Phase × Exercise	0.50	0.487	0.02	0.778
GAS	*Tnf*	Phase	0.31	0.580	0.01	0.580
GAS	*Tnf*	Exercise	1.99	0.170	0.07	0.841
GAS	*Tnf*	Phase × Exercise	1.20	0.284	0.05	0.568
GAS	*Il1b*	Phase	1.41	0.247	0.05	0.396

GAS	*Il1b*	Exercise	0.36	0.551	0.01	0.841
GAS	*Il1b*	Phase × Exercise	2.48	0.128	0.09	0.511
GAS	*Il6*	Phase	2.30	0.142	0.08	0.396
GAS	*Il6*	Exercise	1.07	0.311	0.04	0.841
GAS	*Il6*	Phase × Exercise	0.13	0.718	0.01	0.945
GAS (suppl.)	*Bmal1*	Phase	27.00	<0.0001	0.52	**<0.0001**
GAS (suppl.)	*Bmal1*	Exercise	0.48	0.494	0.02	0.494
GAS (suppl.)	*Bmal1*	Phase × Exercise	0.27	0.605	0.01	0.807
GAS (suppl.)	*Cry1*	Phase	5.17	0.032	0.17	**0.032**
GAS (suppl.)	*Cry1*	Exercise	9.30	0.005	0.27	**0.011**
GAS (suppl.)	*Cry1*	Phase × Exercise	0.02	0.903	0.00	0.903
GAS (suppl.)	*Per2*	Phase	11.79	0.002	0.32	**0.003**
GAS (suppl.)	*Per2*	Exercise	12.15	0.002	0.33	**0.007**
GAS (suppl.)	*Per2*	Phase × Exercise	3.85	0.061	0.13	0.244
GAS (suppl.)	*Nr1d1*	Phase	28.32	<0.0001	0.53	**<0.0001**
GAS (suppl.)	*Nr1d1*	Exercise	1.81	0.191	0.07	0.255
GAS (suppl.)	*Nr1d1*	Phase × Exercise	1.04	0.317	0.04	0.634
SOL	*Nfe2l2*	Phase	2.87	0.103	0.10	0.128
SOL	*Nfe2l2*	Exercise	0.28	0.603	0.01	0.754
SOL	*Nfe2l2*	Phase × Exercise	1.30	0.266	0.05	0.350
SOL	*Hmox1*	Phase	8.36	0.008	0.25	**0.039**
SOL	*Hmox1*	Exercise	6.32	0.019	0.20	0.094
SOL	*Hmox1*	Phase × Exercise	1.56	0.224	0.06	0.350
SOL	*Cat*	Phase	3.61	0.069	0.13	0.123
SOL	*Cat*	Exercise	0.04	0.838	0.00	0.838
SOL	*Cat*	Phase × Exercise	1.22	0.280	0.05	0.350
SOL	*Gpx1*	Phase	0.01	0.937	0.00	0.937
SOL	*Gpx1*	Exercise	0.37	0.549	0.02	0.754
SOL	*Gpx1*	Phase × Exercise	0.28	0.602	0.01	0.602
SOL	*Tnf*	Phase	3.49	0.074	0.12	0.123
SOL	*Tnf*	Exercise	2.04	0.166	0.08	0.414
SOL	*Tnf*	Phase × Exercise	1.93	0.177	0.07	0.350
SOL (suppl.)	*Bmal1*	Phase	4.88	0.037	0.16	**0.037**
SOL (suppl.)	*Bmal1*	Exercise	0.84	0.367	0.03	0.628
SOL (suppl.)	*Bmal1*	Phase × Exercise	1.18	0.287	0.05	0.574
SOL (suppl.)	*Akt1*	Phase	6.44	0.018	0.20	**0.035**
SOL (suppl.)	*Akt1*	Exercise	0.24	0.628	0.01	0.628
SOL (suppl.)	*Akt1*	Phase × Exercise	0.02	0.883	0.00	0.883
QUA	*Nfe2l2*	Phase	6.95	0.014	0.22	0.114
QUA	*Nfe2l2*	Exercise	0.24	0.627	0.01	0.727
QUA	*Nfe2l2*	Phase × Exercise	0.01	0.936	0.00	0.936
QUA	*Sod1*	Phase	0.57	0.459	0.02	0.619
QUA	*Sod1*	Exercise	0.42	0.524	0.02	0.727
QUA	*Sod1*	Phase × Exercise	3.65	0.068	0.13	0.271
QUA	*Cat*	Phase	4.34	0.048	0.15	0.190
QUA	*Cat*	Exercise	0.40	0.532	0.02	0.727
QUA	*Cat*	Phase × Exercise	0.13	0.727	0.00	0.933

QUA	*Gpx1*	Phase	0.58	0.454	0.02	0.619
QUA	*Gpx1*	Exercise	0.16	0.692	0.01	0.727
QUA	*Gpx1*	Phase × Exercise	1.29	0.266	0.05	0.708
QUA	*Tnf*	Phase	0.03	0.856	0.00	0.856
QUA	*Tnf*	Exercise	0.73	0.402	0.03	0.727
QUA	*Tnf*	Phase × Exercise	0.34	0.563	0.01	0.900
QUA	*Il1b*	Phase	0.55	0.465	0.02	0.619
QUA	*Il1b*	Exercise	0.16	0.691	0.01	0.727
QUA	*Il1b*	Phase × Exercise	4.12	0.053	0.14	0.271
QUA	*Il6*	Phase	1.29	0.267	0.05	0.619
QUA	*Il6*	Exercise	2.94	0.099	0.11	0.727
QUA	*Il6*	Phase × Exercise	0.06	0.816	0.00	0.933
QUA	*Il10*	Phase	0.12	0.733	0.00	0.838
QUA	*Il10*	Exercise	0.13	0.727	0.00	0.727
QUA	*Il10*	Phase × Exercise	0.89	0.354	0.03	0.708
Liver	*Nfe2l2*	Phase	0.06	0.816	0.00	0.974
Liver	*Nfe2l2*	Exercise	0.70	0.412	0.03	0.885
Liver	*Nfe2l2*	Phase × Exercise	0.00	0.975	0.00	0.975
Liver	*Keap1*	Phase	18.75	<0.001	0.43	**0.001**
Liver	*Keap1*	Exercise	0.02	0.885	0.00	0.885
Liver	*Keap1*	Phase × Exercise	4.97	0.035	0.17	0.245
Liver	*Tnf*	Phase	0.00	0.974	0.00	0.974
Liver	*Tnf*	Exercise	0.50	0.488	0.02	0.885
Liver	*Tnf*	Phase × Exercise	0.82	0.373	0.03	0.653
Liver	*Il1b*	Phase	0.60	0.447	0.02	0.974
Liver	*Il1b*	Exercise	0.05	0.825	0.00	0.885
Liver	*Il1b*	Phase × Exercise	0.01	0.943	0.00	0.975
Liver	*Il6*	Phase	0.10	0.758	0.00	0.974
Liver	*Il6*	Exercise	0.25	0.622	0.01	0.885
Liver	*Il6*	Phase × Exercise	0.08	0.778	0.00	0.975
Liver	*Ccl2*	Phase	0.00	0.972	0.00	0.974
Liver	*Ccl2*	Exercise	0.04	0.842	0.00	0.885
Liver	*Ccl2*	Phase × Exercise	2.63	0.117	0.10	0.274
Liver	*Adgre1*	Phase	0.57	0.459	0.02	0.974
Liver	*Adgre1*	Exercise	1.06	0.313	0.04	0.885
Liver	*Adgre1*	Phase × Exercise	3.58	0.070	0.13	0.245

Two-way analysis of variance (ANOVA) was performed with circadian phase (ZT3 vs. ZT15) and exercise status (sedentary vs. exercise) as factors (ZT3-sed $n = 5$, ZT3-exe $n = 8$, ZT15-sed $n = 8$, ZT15-exe $n = 8$). The Benjamini–Hochberg false discovery rate (FDR) procedure was applied separately for each ANOVA term (phase, exercise, and phase × exercise) within each outcome family: plasma redox markers, the gene panel of each tissue, and the supplementary genes (suppl.) in gastrocnemius (GAS) and soleus (SOL). Bold q values indicate $q < 0.05$. p, unadjusted p value; ηp^2, partial eta squared; q, FDR-adjusted p value; OXY, OXY-adsorbent test; d-ROMs, derivatives of reactive oxygen metabolites test; BAP, biological antioxidant potential test; QUA, quadriceps.

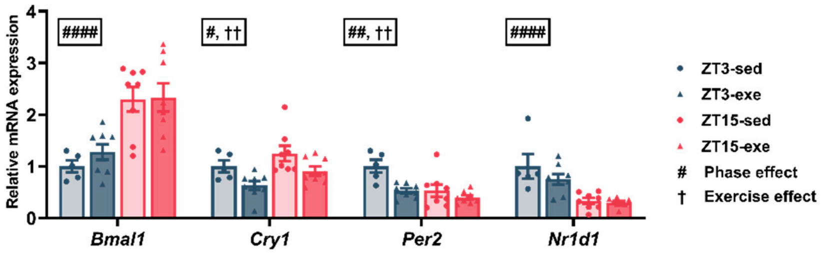

Figure S1. Core clock gene expression in GAS.

Core clock genes (*Bmal1*, *Cry1*, *Per2*, and *Nr1d1*). Statistical analysis was performed using two-way analysis of variance (ANOVA, phase × exercise). Data are means ± standard error of the mean (SEM, ZT3-sed $n = 5$, ZT3-exe $n = 8$, ZT15-sed $n = 8$, ZT15-exe $n = 8$; sed, sedentary; exe, exercise). # indicates a phase main effect, and † indicates an exercise main effect; # $p < 0.05$, ##/†† $p < 0.01$, #### $p < 0.0001$. Symbols indicate unadjusted p values; FDR-adjusted q values are provided in Table S2.

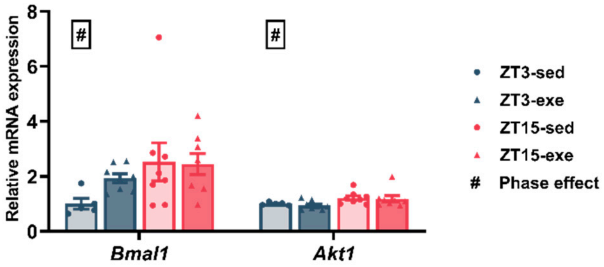

Figure S2. *Bmal1* and *Akt1* expression in SOL.

Bmal1 and *Akt1*. Statistical analysis was performed using two-way ANOVA (phase × exercise). Data are means ± SEM (ZT3-sed $n = 5$, ZT3-exe $n = 8$, ZT15-sed $n = 8$, ZT15-exe $n = 8$). # indicates a phase main effect. # $p < 0.05$. Symbols indicate unadjusted p values; FDR-adjusted q values are provided in Table S2.
